# Supplementary material for: Proteomic associations with cognitive variability as measured by the Wisconsin Card Sorting Test in a healthy Thai population: A machine learning approach
Source: PLoS One. 2025 Feb 20;20(2):e0313365. doi: 10.1371/journal.pone.0313365 (PMC11841870; doi:10.1371/journal.pone.0313365)
Supplement: S1 File — (DOCX) [file pone.0313365.s002.docx]

> library(DMwR)

> library(tidymodels)

> set.seed(001)

> split_pbp <- initial_split(my_data,prop = 0.60,strata = risk_group)

> train_data <- training(split_pbp)

> test_data <- testing(split_pbp)

> table(train_data$risk_group)

> table(test_data$risk_group)

> new_train <- SMOTE(risk_group~., train_data, perc.over = 4000, perc.under = 400)

> table(new_train$risk_group)

> tune_spec <- rand_forest(

mtry = tune(),

trees = 1000,

min_n = tune()

) %>%

set_mode("classification") %>%

set_engine("ranger",importance = "permutation")

> tune_wf <- workflow() %>%

add_model(tune_spec) %>%

add_formula(risk_group~.)

> set.seed(234)

> trees_folds <- vfold_cv(new_train)

> rf_grid <- grid_regular(

mtry(range = c(10, 30)),

min_n(range = c(2, 8)),

levels = 5

)

> set.seed(456)

> regular_res <- tune_grid(

tune_wf,

resamples = trees_folds,

grid = rf_grid

)

> best_auc <- select_best(regular_res, "roc_auc")

> final_rf <- finalize_model(

tune_spec,

best_auc

)

> final_rf

> final_wf <- workflow() %>%

add_model(final_rf) %>%

add_formula(risk_group~.)

> final_res <- final_wf %>%

last_fit(split_pbp)

> final_res %>%

collect_metrics()

> fit_rf <- final_wf %>%

fit(new_train)

> pred_rf <- test_data %>% select(risk_group) %>%

bind_cols(predict(fit_rf, test_data, type = "prob")) %>%

bind_cols(predict(fit_rf, test_data, type = "class"))

>

> metricsets <- metric_set(accuracy, mcc, f_meas, j_index)

> pred_rf %>% metricsets(truth = risk_group, estimate = .pred_class)

> pred_rf %>% conf_mat(truth = risk_group, estimate = .pred_class)

> pred_rf %>% conf_mat(risk_group,.pred_class) %>%

autoplot()

> pred_rf %>% roc_auc(risk_group, .pred_High)

> pred_rf %>% roc_curve(truth = risk_group, .pred_High) %>%

autoplot()

> library(vip)

> fit_rf %>%

extract_fit_parsnip() %>%

vip(num_features = 10)

> rf_fit_rs <-

final_wf %>%

fit_resamples(trees_folds)

>

> collect_metrics(rf_fit_rs)

> rf_spec <- rand_forest(trees = 1000,mtry = 10,min_n = 2) %>%

set_engine("ranger",importance = "permutation") %>%

set_mode("classification")

> rf_tuned_wf <- workflow() %>%

add_model(rf_spec) %>%

add_formula(risk_group~.)

>

> fit_tuned_rf <- rf_tuned_wf %>%

fit(new_train)

>

> fit_tuned_rf %>%

extract_fit_parsnip() %>%

vip(num_features = 10)

>

> pred_tuned_rf <- test_data %>% select(risk_group) %>%

bind_cols(predict(fit_tuned_rf, test_data, type = "prob")) %>%

bind_cols(predict(fit_tuned_rf, test_data, type = "class"))

> pred_tuned_rf %>% metricsets(truth = risk_group, estimate = .pred_class)

> pred_tuned_rf %>% conf_mat(truth = risk_group, estimate = .pred_class)

> pred_tuned_rf %>% roc_auc(risk_group, .pred_High)

> pred_tuned_rf%>% conf_mat(risk_group,.pred_class) %>%

autoplot()

> pred_rf %>% roc_curve(truth = risk_group, .pred_High) %>%

autoplot()
